# Microbial lipases: advances in production, purification, biochemical characterization, and multifaceted applications in industry and medicine

**DOI:** 10.1186/s12934-025-02664-6

**Published:** 2025-02-12

**Authors:** Ahmed A. Abdelaziz, Amal M. Abo-Kamar, Esraa Sobhy Elkotb, Lamiaa A. Al-Madboly

**Affiliations:** Department of Microbiology and Immunology, Faculty of Pharmacy, Tanta University, Tanta, Egypt

**Keywords:** Lipases, Biocatalysts, Microbial lipases, Enzyme production, Fermentation strategies, Purification techniques, Enzyme stability, Agro-industrial residues, Bioremediation, Biofuels, Cosmetics, Biodegradation, Biomedical applications, Drug delivery, Enzyme technology

## Abstract

Lipases are biocatalysts of significant industrial and medical relevance, owing to their ability to hydrolyze lipid substrates and catalyze esterification reactions under mild conditions. This review provides a comprehensive overview of microbial lipases’ production, purification, and biochemical properties. It explores optimized fermentation strategies to enhance enzyme yield, including using agro-industrial residues as substrates. The challenges associated with purification techniques such as ultrafiltration, chromatography, and precipitation are discussed, alongside methods to improve enzyme stability and specificity. Additionally, the review addresses the growing importance of genetic engineering approaches for improving lipase characteristics, such as activity, stability, and specificity.

Additionally, this review highlights the diverse applications of microbial lipases in industries, including food, pharmaceuticals, biofuels, and cosmetics. The enzyme’s role in bioremediation, biodegradation, and the synthesis of bioactive compounds is analyzed, emphasizing its potential in sustainable and eco-friendly technologies. The biocatalytic properties of lipases make them ideal candidates for the green chemistry initiatives in these industries. In the biomedical domain, lipase has shown promise in drug delivery systems, anti-obesity treatments, and diagnostics.

This review provides insights into the strategic development of microbes as microbial cell factories for the sustainable production of lipases, paving the way for future research and industrial innovations in enzyme technology.

## Introduction

Enzymes serve as biocatalysts in all living organisms, characterized by their remarkable ability to facilitate specific reactions with minimal energy expenditure. They are integral to various metabolic processes and biochemical reactions. Certain enzymes are particularly noteworthy due to their potential as catalysts in diverse biochemical applications. Among these, lipases, a subclass of esterases, play a crucial role in the digestion, transport, and processing of lipids across most living organisms. Their versatility allows them to catalyze a variety of bioconversion reactions, including hydrolysis, alcoholysis, acidolysis, aminolysis, esterification and interesterification, in both unicellular and multicellular entities. Lipases are vital for the bioconversion of triacylglycerols (TAG) between organisms and within individual organisms. Moreover, lipases exhibit a unique capability to function at the interface of aqueous and non-aqueous phases, setting them apart from other esterases. Additional distinctive characteristics of lipases encompass specificity, pH sensitivity, temperature responsiveness, catalytic efficiency in organic solvents, and their non-toxic nature. These features make them ideal for a wide range of industrial applications, where traditional catalysts may fail.

The most advantageous attributes of lipases include their proficiency in processing various glycerides (mono-, di-, and triglycerides) and free fatty acids during transesterification, their exceptional activity in non-aqueous environments, minimal product inhibition, and resilience to fluctuations in temperature and pH. Furthermore, lipases demonstrate stability in organic solvents and maintain activity without the need for cofactors. These enzymes are ubiquitous in all living organisms and are essential for their normal physiological functions. The application of microbial lipases derived from fungi, yeast, and bacteria has garnered significant interest in recent decades [1].

Microbial lipases are considered more valuable than those derived from plants or animals due to their diverse catalytic activities, high production yields, and ease of genetic manipulation. They are not subject to seasonal variations, ensuring a consistent supply, and they exhibit greater stability, safety, and convenience. Additionally, microorganisms demonstrate a significantly high growth rate in economically viable media [2]. The scalability of microbial lipase production further adds to its industrial relevance, making it a preferred choice for large-scale processes [3]. Bacterial isolates are known for their enhanced activities, particularly at neutral or alkaline pH levels, and their thermostability, which is also associated with certain yeasts [4]. Notable bacterial strains include *Pseudomonas alcaligenes*,* P. aeruginosa*,* P. fragi*,* P. fluorescens BJ-10*,* Bacillus subtilis*,* and B. nealsonii S2MT*, along with various fungal species such as Penicillium expansum,* Trichoderma*,* and Penicillium chrysogenum*. Lipases derived from microbial sources exhibit unique physiochemical and biological properties that enhance their role as significant biocatalysts, demonstrating effectiveness as viable alternatives to traditional organic methods for the selective transformation of complex molecules across various industries [2]. This review also emphasizes the importance of exploring new microbial strains that could yield lipases with improved characteristics for specific applications.

In the food sector, lipases are widely utilized and contribute significantly to the production of diverse food items, including baked goods, juices, and fermented products. Additionally, lipases find extensive applications in industrial cleaning, leather processing, cosmetics, paper manufacturing, and the detergent industry. Their relevance extends to other fields such as biosensors, biodiesel production, biomedical applications, pesticides, and bioremediation, highlighting their importance. In terms of total sales, lipases rank among the largest enzyme groups, with the global lipase market projected to exceed USD 797.7 million by 2025 [5].

## History of lipase

The discovery of lipase dates back to 1848 when Claude Bernard, often considered the father of modern physiology, identified pancreatic lipase. After 22 unsuccessful attempts, Bernard isolated pancreatic juice from a dog and added tallow from a candle to the secretion. He observed the emulsification of the fat, which led him to conclude that lipase was present in the pancreas. Today, it is well understood that pancreatic lipase plays a crucial role in the digestion and absorption of fats in the human body [6].

## Properties and characteristics of lipases

Lipases generally have a molecular weight between 19 and 60 kDa and are primarily classified as monomeric proteins. Their characteristics are shaped by various factors, including the positioning of fatty acids on the glycerol backbone, the length of the fatty acid chains, and their level of unsaturation [2]. These factors also influence the sensory and nutritional qualities of the associated triglycerides. Different lipases enable a variety of beneficial reactions, such as esterification, due to their activity in organic solvents [7].

The activity of lipases is dependent on pH, with optimal stability typically found at a neutral pH of 7.0, and they remain stable within a pH range of 4.0 to 8.0. Extracellular lipases produced by organisms like *Chromobacterium viscosum*,* Aspergillus niger*, and various *Rhizopus* species demonstrate activity in acidic environments, while *Pseudomonas nitroaeruginosa* produces an alkaline lipase that functions effectively at pH 11.0 [8].

Although cofactors are not required for lipase activity, the presence of calcium, a divalent cation, can enhance their performance [12]. In contrast, metals such as Co, Ni²⁺, Hg²⁺, and Sn²⁺ significantly inhibit lipase activity, while Zn²⁺, Mg²⁺, EDTA, and SDS have a less pronounced inhibitory effect. The half-life values of lipases reflect their thermal stability, with lower temperatures generally offering greater stability [9].

Lipases are categorized into two groups based on their region-specific characteristics, particularly in relation to acyl glycerol substrates. In the first group of lipases, fatty acids are released from all three positions of glycerol without any regiospecificity [14]. Conversely, the second group of lipases exhibits regiospecificity, selectively releasing fatty acids from the 1 and 3 positions of acylglycerols. These lipases hydrolyze triacylglycerols, resulting in the formation of 2-monoacylglycerol and free fatty acids, as well as 1,2-(2,3)-diacylglycerols. Partial stereospecificity has been observed in the hydrolysis of triacylglycerols in species such as *A. arrhizus*,* R. delemar*,* C. cylindracea*, and *P. aeruginosa* [10]. These enzymatic properties make them suitable for the extraction of optically pure esters and alcohols. Furthermore, utilizing organic media at low water activity presents a significant opportunity for solvent variation. By altering solvent properties, the specificity of enzymes can be modified, as solvents can substantially influence the catalytic behaviour of enzymes due to their soft and delicate structures [11].

## Sources for microbial lipases

Microbial lipases are ubiquitous in nature and hold significant commercial value due to their low production costs, enhanced stability, and greater availability compared to lipases derived from animals and plants Table 1. Both naturally occurring and recombinant microbial lipases are widely utilized in various bioengineering applications [12]. The vast array of microbial resources available in nature demonstrates their remarkable adaptability to extreme environments, such as the Dead Sea, Antarctica, alkaline lakes, hot springs, volcanic vents, and contaminated soils, which presents exceptional opportunities for the production of lipases with unique characteristics [2]. A substantial advancement in enantioselective hydrolysis and the synthesis of carboxyl esters has led to increased accessibility of these enzymes. Marine microflora, in particular, exhibit enhanced capabilities for producing active enzymes and proteins. Predominantly, lipases are secreted extracellularly by fungi and bacteria. In various biocatalytic applications, *Candida antarctica* lipase B (CALB) is the most frequently utilized enzyme and holds a significant number of patents. Another important lipase derived from yeast is *C. rugosa* lipase (CRL), which consists of a blend of different isoforms and is commercially available. This enzyme is classified as “Generally Recognized As Safe” (GRAS) and is employed in the food industry [13]. Phospholipases A1 and A2 from *Fusarium oxysporum*, *T. lanuginosus*, *A. niger*, and *Trichoderma reesei* are utilized in the degumming of vegetable oils and are commercially produced. Among these, PLA1s, PLA2s, and PLBs extracted from *A. oryzae* and *A. niger* are predominantly used in the food sector. Additionally, phospholipase D (PLD) enzymes isolated from Actinomycete strains are commercially available due to their high transphosphatidylation and hydrolytic activities, finding applications in various industrial processes [14].

**Table 1:** A Comparative Assessment of Plant, Animal, and Microbial Variants Based on Cost, Efficiency, and Stability [15]

Source	Cost	Efficacy	Stability
Plant-Based	Inexpensive raw materials but extraction and purification increase costs.	Moderate activity; limited substrate diversity.	Less stable under extreme pH and temperature conditions
Animal-Based	Costly due to complex extraction and ethical considerations.	High substrate specificity; requires specific conditions.	Stable within narrow pH and temperature ranges; less versatile for broader industrial uses.
Microbial-Based	Cost-effective; high yield via fermentation and low-cost substrates.	Highly efficient; broad substrate range and versatile for diverse applications.	Exceptionally stable under extreme conditions (pH, temperature); enhanced through genetic engineering.

### Bacterial lipases

**Fig. 1:**
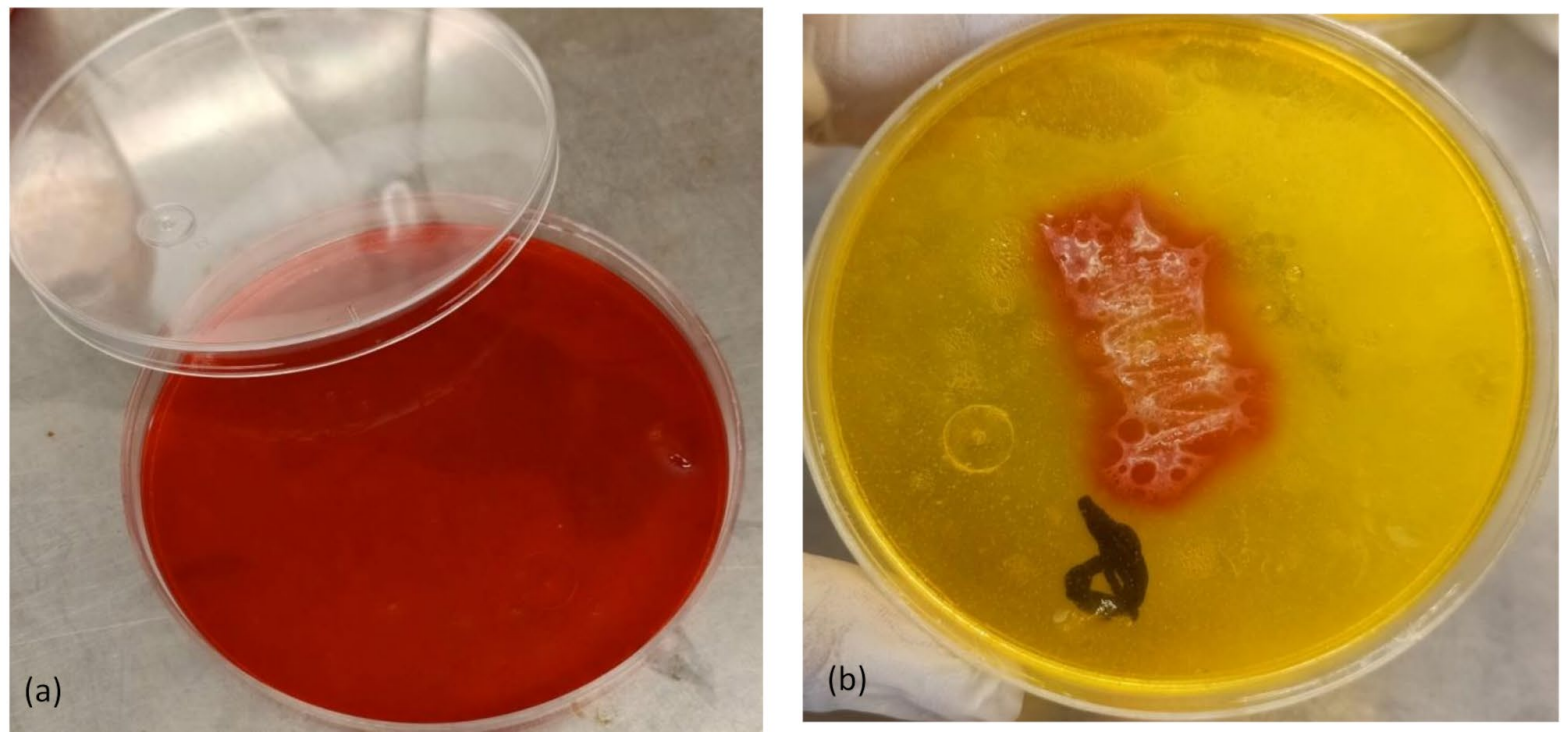
The production of lipase enzyme from *P. aeruginosa* isolate using olive oil as a substrate in the phenol red agar (**a**) control medium showed the red color before growth, (**b**) showed growth of the microorganism at the center of the medium and change in the color of the pH-indicator (phenol red) into the yellow color due to lipase production

Bacterial lipases were first identified in 1901 in species such as *B. prodigiosus* and *B. fluorescens*. Currently, *Serratia marcescens* and *P. aeruginosa* as in Fig. (1) are recognized as leading producers of lipases among bacteria. These enzymes are classified as glycoproteins and lipoproteins. The production of lipases in many bacterial species is influenced by specific polysaccharides [16].

### Fungal lipases

Since the 1950s, research on fungal lipases has gained momentum due to their remarkable thermal and pH stability, substrate specificity, and effectiveness in organic solvents and downstream processing applications [17]. These characteristics have led to their widespread utilization. In contemporary practices, batch fermentation and cost-effective extraction methods are preferred, providing fungal lipases with advantages over bacterial alternatives. Notable filamentous fungal genera involved in lipase production include *Rhizopus*, *Aspergillus*, *Penicillium*, Mucor, *Ashbya*, *Geotrichum*, *Beauveria*, *Humicola*, *Rhizomucor, Fusarium, Acremonium*, Alternaria, *Eurotrium*, and *Ophiostoma*. Additionally, species such as *C. rugosa*,* C. antarctica*,* T. lanuginosus*,* Rhizomucor miehei*, *Pseudomonas*, *Mucor*, and *Geotrichum* are also significant contributors [18]. Among these, Colletotrichum gloesporioides has been identified as the most productive strain, yielding 27,700 U/l of lipase from Brazilian savanna soil through enrichment culture techniques. The primary commercial producers of these lipases include *A. niger*,* C. rugosa*,* H. lanuginosa*,* M. miehei*,* R. arrhizus*,* R. delemar*,* R. japonicus*,* R. niveus*, and *R. oryzae* [17].

## Production and optimization of lipase enzyme

Lipase enzymes, which catalyze the hydrolysis of ester bonds in lipids, are of significant industrial importance, especially in the food, pharmaceutical, and biofuel industries. The production of lipase enzymes is typically carried out through microbial fermentation, where microorganisms such as bacteria, fungi, and yeast are cultured under specific conditions to generate high levels of the enzyme. The most common methods for lipase production include submerged fermentation (SmF), solid-state fermentation (SSF), and the use of genetically modified organisms (GMOs) [3].

**Fig. 2:**
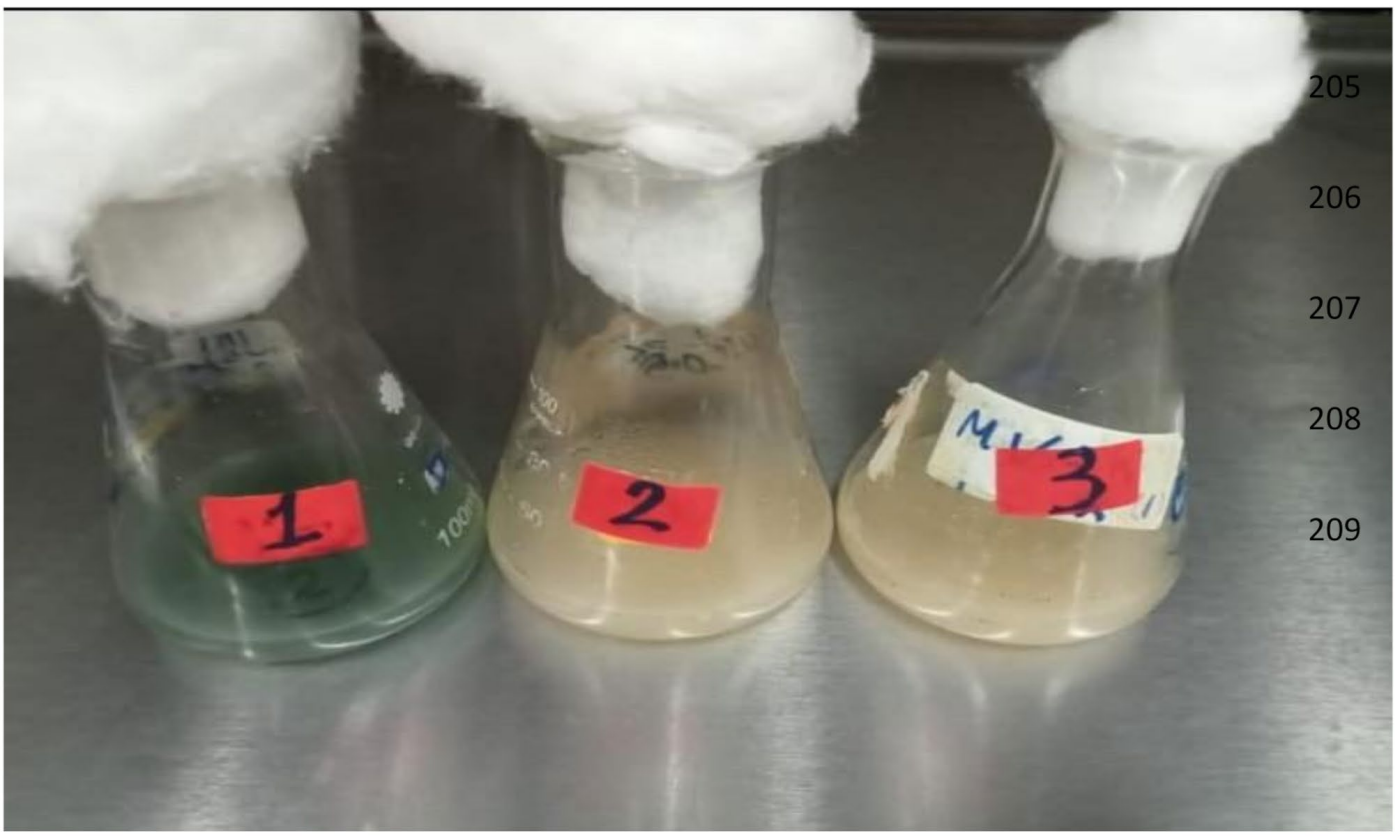
Production of lipase enzyme from *P. aeruginosa* strain by submerged fermentation process using ethidium bromide for random mutagenesis

Submerged fermentation (SmF) is one of the most widely used methods for lipase production. In this process, microorganisms are cultured in a liquid medium, and lipase is secreted into the solution as in Fig. (2). This method is scalable and offers the advantage of easier control over variables such as temperature, pH, and oxygen supply. In contrast, solid-state fermentation (SSF) involves the cultivation of microorganisms on solid substrates like wheat bran or rice husk with minimal moisture. SSF is considered more cost-effective and has been shown to result in higher lipase activity in certain cases due to the solid-state environment promoting the enzyme’s production. The choice between SmF and SSF often depends on the specific requirements of the industrial application Table 2, including cost considerations and the desired enzyme yield [18, 19].

**Table 2:** Comparative analysis of submerged fermentation (SmF) and solid-state fermentation (SSF) in Industrial Applications [20]

Factor	Submerged Fermentation (SmF)	Solid-State Fermentation (SSF)
Cost	Generally higher due to energy requirements for aeration and agitation, and the use of liquid substrates.	Lower cost as it uses cheaper solid substrates, and energy consumption is lower.
Yield	Typically higher yields due to better control over temperature, pH, and nutrient availability.	Lower yields compared to SmF but can be enhanced through optimized conditions.
Industrial Suitability	Suitable for large-scale production of liquid products (enzymes, biofuels, etc.). Needs advanced equipment and infrastructure.	More suitable for production of solid products (e.g., enzymes, mushrooms) and often used in low-tech settings or small-scale production.
Process Control	Easier to control parameters like pH, temperature, and oxygen levels.	Harder to control parameters as solid substrates limit oxygen diffusion and moisture content control.
Microbial Growth	Promotes rapid microbial growth due to high moisture content and aeration.	Microbial growth is slower and limited by oxygen and moisture availability in the solid substrate.
Applications	Enzyme production, biofuel production, antibiotics, and other liquid fermentation products.	Production of solid enzymes, biopesticides, fermented foods, and biofuels.

Genetically modified organisms (GMOs) have also been explored to improve lipase production (Fig. 2). Through genetic engineering, microorganisms can be altered to overexpress specific genes involved in lipid metabolism, significantly enhancing lipase yield. This method leverages biotechnological advancements to create strains with superior lipase-producing capabilities. The use of GMOs can further optimize microbial fermentation by enabling microorganisms to withstand adverse conditions and produce lipase more efficiently [21].

Agro-industrial waste, such as olive mill waste, rice husk, and sugarcane bagasse, has potential for lipase production but faces logistical challenges. The high moisture content and volume of these wastes complicate storage and transportation, requiring specialized facilities. Pre-treatment processes like enzymatic hydrolysis are often necessary to make the waste suitable for microbial growth. Despite these challenges, agro-wastes can be efficient substrates for lipase production when optimized fermentation conditions are used. The environmental impact is significant, as using agro-industrial waste reduces pollution, minimizes landfill waste, and lowers the carbon footprint compared to conventional fuels. However, the environmental benefits depend on maintaining an energy-efficient balance in waste processing and bioproduct generation [22].

The transition of lipase production from laboratory scale to industrial scale involves several key challenges related to infrastructure and cost. On the infrastructure side, the primary considerations include the design of bioreactors, as the shift from small-scale laboratory fermenters to large industrial reactors requires precise control over variables such as temperature, aeration, and agitation. At the industrial scale, automated control systems are essential to regulate parameters like pH, temperature, and feeding times to ensure consistent enzyme production across larger batches. Furthermore, the increase in production volume demands efficient mixing and nutrient distribution to maintain optimal conditions for microbial growth and enzyme synthesis [23].

In terms of cost challenges, raw material expenses significantly increase at the industrial scale, as larger quantities of carbon and nitrogen sources are required. Additionally, energy consumption escalates, particularly in larger bioreactors, demanding more efficient energy usage. To overcome these challenges, several strategies can be employed, such as the use of cost-effective raw materials, including recycled oils or by-products from other industries [24]. Another approach is optimizing fermentation processes using techniques like Response Surface Methodology (RSM), which can enhance raw material consumption and minimize waste. Economic modeling also helps in identifying cost-effective scaling methods, optimizing resource allocation, and maintaining high profitability [25].

To address infrastructure challenges, investment in modern equipment is crucial, including large-scale fermenters and advanced logistical systems to streamline material handling and product distribution. Improved bioreactor designs, such as those with dual impellers, can ensure uniform nutrient and oxygen distribution, enhancing fermentation efficiency. Managing waste efficiently and recycling by-products helps minimize costs, while technological advancements in reactor design improve both scale-up performance and cost-effectiveness [23].

Optimization of lipase production is a key step in maximizing enzyme yields while minimizing production costs. Several factors influence lipase production, including the type of carbon and nitrogen sources, temperature, pH, and the presence of inducers. Optimization techniques, such as the use of response surface methodology (RSM), are commonly employed to identify the most favourable conditions for lipase production [26]. It is a powerful statistical tool that facilitates the optimization of multiple variables simultaneously. It employs mathematical models and experimental designs, such as Central Composite Design (CCD) or Box-Behnken Design, to analyze the interactions between factors and predict the optimal conditions. RSM reduces the number of experimental runs needed, saving time and resources while providing precise insights into the relationships between key variables. In a study by Lo, C.-F., et al. (2012) [27], RSM was employed to optimize the production of lipase by a bacterial strain. Factors such as carbon source, nitrogen source, and substrate concentration were evaluated, leading to increasing of activity three-fold higher than that in basal medium.

The choice of carbon and nitrogen sources plays a significant role in the optimization of lipase production. Carbon sources like oils (e.g., olive oil or sunflower oil) serve as both a carbon source and an inducer of lipase activity. Nitrogen sources such as yeast extract or ammonium salts are crucial for promoting microbial growth and facilitating enzyme secretion. The optimization of these nutritional factors helps in maximizing lipase production by providing the microorganisms with the necessary elements for growth and enzyme synthesis. Additionally, the addition of specific inducers such as fatty acids or oils can further enhance lipase activity, as these compounds stimulate the metabolic pathways responsible for enzyme production [28].

Temperature and pH are also critical factors that affect lipase production. Each microorganism has an optimal temperature and pH range for maximum enzyme secretion, and even small variations in these parameters can significantly impact lipase yield. For example, certain microbial strains may produce higher lipase levels at slightly elevated temperatures or under mildly acidic conditions. Therefore, the careful regulation of these factors is necessary to ensure optimal enzyme production [29].

### Practical relevance of RSM

In addition to laboratory-scale studies, RSM has shown significant potential in industrial-scale applications. By optimizing operational parameters, industries can achieve cost-effective and scalable production of lipase, enhancing overall process efficiency. Furthermore, combining RSM with other modern techniques such as machine learning or genetic optimization opens new avenues for further improving lipase production processes [30].

In recent years, metabolic engineering and genetic modification have been increasingly used to improve lipase production. By overexpressing specific genes involved in lipid metabolism or introducing foreign genes, genetically modified strains can be designed to produce higher quantities of lipase. Metabolic engineering also allows for the optimization of cellular pathways, resulting in enhanced enzyme yields. The use of genetically modified microorganisms in combination with traditional optimization methods provides a promising approach for large-scale industrial lipase production [31].

## Purification strategies of microbial lipases

The purification of microbial lipases is a precise process that necessitates careful handling to preserve the enzyme’s bioactive form. Various microbial sources have yielded lipases that have been purified to a high degree of homogeneity through a range of techniques. These purification methods can be categorized into two main types: classical purification methods and modern purification methods. Classical methods tend to be non-specific, labor-intensive, and involve multiple steps, often resulting in insufficient purity. In contrast, modern purification methods are more efficient, specific, scalable, and capable of achieving elevated purity levels. Advances in purification strategies have facilitated the development of specialized techniques tailored for different microbial lipases. Achieving a homogeneous state in lipase purification has enabled the determination of amino acid sequences and their three-dimensional structures, thereby enhancing the understanding of their distinctive properties in various biochemical reactions [32].

### Traditional purification methods

#### Precipitation and chromatographic techniques

**Fig. 3:**
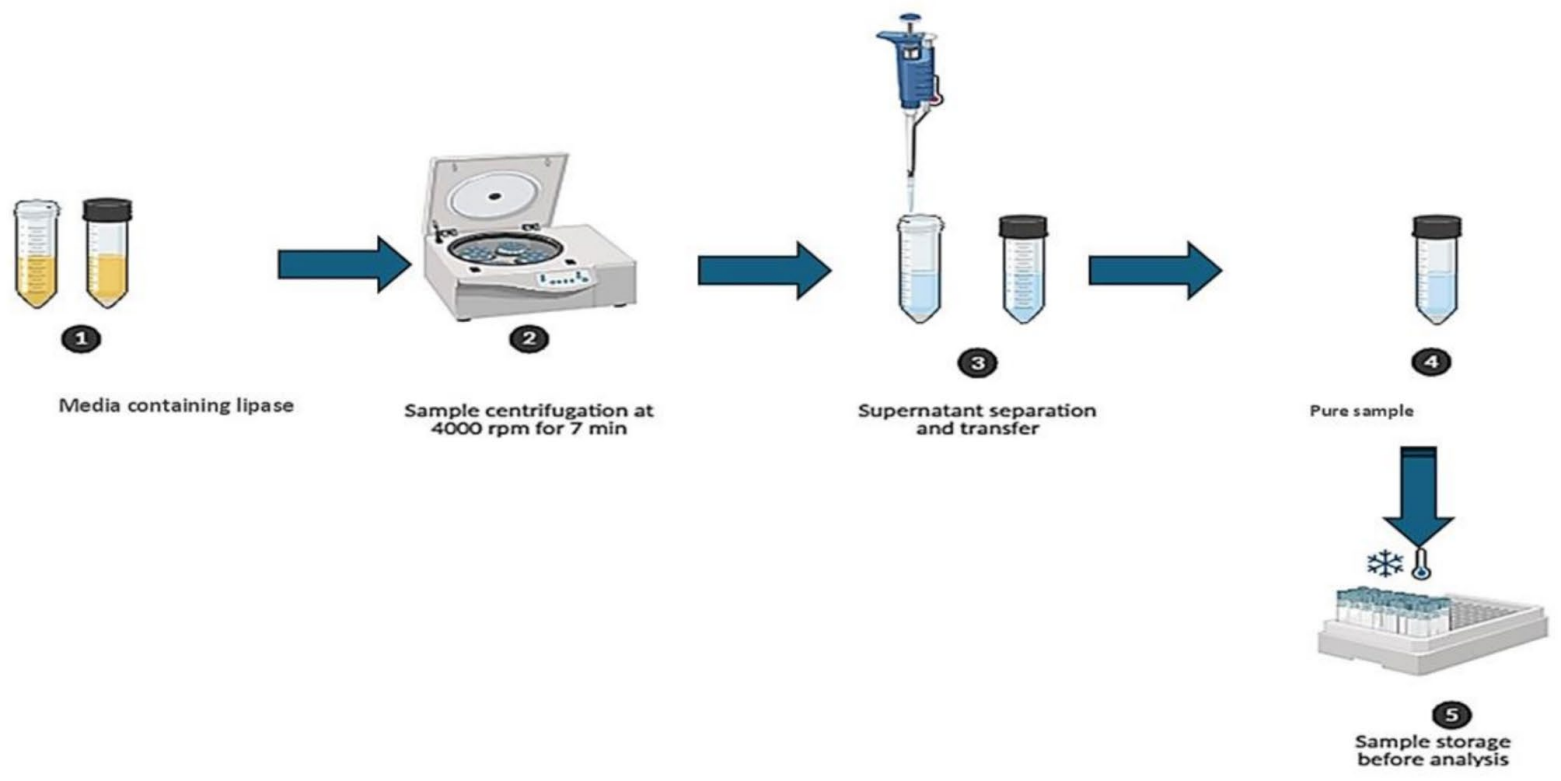
Schematic diagram showing the steps of lipase production and purification from bacterial strain until obtaining a pure lipase extract [36]

Initially, the purification of lipases from culture broth was achieved through filtration or centrifugation methods. Microbial lipases are predominantly extracellular, necessitating the separation of cells from the culture medium following fermentation. The purification process typically begins with precipitation as in Fig. (3), which is subsequently followed by chromatographic separation techniques. Nearly all purification protocols incorporate a precipitation step, with ammonium sulfate, ethanol, acetone, or hydrochloric acid being the most frequently utilized agents. Literature indicates that precipitation has been employed in over 80% of purification strategies, with ammonium sulfate being used twice as often (60%) as ethanol or acetone (30%) [33]. A study by Liu et al. [34] also employed ammonium sulfate precipitation followed by ion-exchange chromatography and gel filtration to purify lipase from *Aspergillus niger* AN0512, achieving aproximately 204-fold purification with 22.1% recovery.

Another investigation by Rabbani et al. [35] The purification steps included ammonium sulfate precipitation and ion-exchange chromatography, resulting in a 10-fold increase in activity compared to the standard enzyme.

The precipitated enzymes were subsequently purified using a dialysis membrane. While lipase activity increased with higher concentrations of ammonium sulfate, the study also indicated that saturating ammonium sulfate beyond 80% (w/v) inhibited activity. Notably, precipitation methods yielded an average of 87%, which is significantly higher than that of other techniques [37]. Furthermore, the purification of halophilic lipases was reported to involve a three-step process that included ammonium sulfate precipitation followed by two rounds of ion-exchange chromatography [38]. The choice of the suitable chromatographic technique is contingent upon the specific lipase preparation and the overall purification strategy. Commonly utilized ion exchangers include the diethyl amino ethyl (DEAE) group for anion exchange and the carboxy methyl (CM) group for cation exchange, which is often followed by gel filtration purification [39]. Contemporary purification methods are implemented to enhance lipase yield and minimize processing duration. Advances in purification techniques have expanded the options available for designing tailored purification protocols for microbial lipases [40].

### Contemporary purification techniques

The benefits of contemporary purification techniques are largely attributed to their superior yields and reduced processing times compared to traditional methods. Below, we present examples of contemporary purification techniques that have been discussed in recent review articles, focusing on their practical applications and commercial significance Table 3.

#### Reverse micellar systems (RMS)

Reverse micelles are conceptualized as microreactors that provide a protective environment for enzymes against the harmful effects of solvents. The architecture of a reverse micelle features an aqueous microdomain that is oriented towards the polar heads of the surfactants, which encase this core and engage with the surrounding non-polar organic solvent via their hydrophobic tails [41]. The reverse micellar system (RMS) operates through two critical phases: forward extraction and reverse extraction. For example, Gaikaiwari et al. [42] utilized both traditional and reverse micellar methods to purify lipase from *Pseudomonas* strains, achieving a 15-fold enhancement in purification efficiency with an 80% recovery rate in just 45 min, while the conventional method resulted in only 52% enzyme recovery over a period of 30 to 40 h. This highlights the advantages of RMS in terms of time and efficiency. During the forward extraction phase, lipase is transferred from an aqueous environment to an organic phase, where it becomes encapsulated within reverse micelles, thereby safeguarding it from denaturation upon exposure to the organic solvent. Conversely, in the reverse extraction phase, the lipase is not retained within the reverse micelles for an extended duration; instead, it is efficiently moved back into an aqueous medium without contamination. Recently, surfactants have become commonplace in RMS applications, as they improve the solubility of organic compounds and lower surface tension [43]. The structure of surfactants, characterized by a hydrophilic head group linked to a hydrophobic tail, closely mimics the natural substrates of lipases. This similarity allows surfactants to be utilized in capturing the open conformation of lipases during crystallization investigations. For example, the open conformation of a member of the thermoalkalophilic lipase family was observed in the presence of non-ionic surfactants [44].

In reverse micellar systems (RMS), surfactants increase the interfacial area of the solvent, promoting micelle formation and enhancing the recovery of enzymes from the system. This role of surfactants, combined with micelle formation, leads to more efficient downstream processing and improved enzyme purification [45]. This technique has shown its potential in improving enzyme yields in industrial applications by enabling high enzyme recovery and reducing purification time, thus contributing to cost efficiency [46]. Gaikaiwari et al. [42] utilized both traditional and reverse micellar methods to purify lipase from *Pseudomonas*. Their results showed that the RMS approach achieved a 15-fold enhancement in purification efficiency with an 80% recovery rate in just 45 min, whereas the conventional method resulted in only 52% enzyme recovery over a period of 30 to 40 h. The RMS technique emerged as the fastest and most cost-effective option for purifying lipase from bacterial sources. Furthermore, Fernandes et al. [47] demonstrated the efficacy of lipase from the thermophilic fungus *Thermomyces lanuginosus* in hydrolysis and synthesis reactions within an RMS, achieving a high yield of 200 U/mg in a relatively short period.

#### Immunopurification

Immunopurification refers to the use of affinity chromatography to isolate a target protein through an antibody-antigen interaction. This innovative method, also known as immunoaffinity chromatography, is effective for the precise separation of specific proteins. In this process, both affinity-purified polyclonal and monoclonal antibodies are employed to isolate the target proteins. Initially perceived as a costly approach, advancements in the mass production of antibodies have facilitated the industrial application of immunopurification utilizing various monoclonal antibodies [48]. The industrial adaptation of immunopurification techniques has significantly improved with the development of more cost-effective monoclonal antibody production, making it a viable option for large-scale enzyme purification. Research conducted by Rahimi et al. [49] discovered two monoclonal antibodies (MoAbs), BF11 and VNH9, that successfully immobilize lipase from *C. rugosa*. Both antibodies, which are of the IgG1 isotype, recognize specific antigenic determinants common to various isoforms of *C. rugosa* lipase. After the immune complex was formed, the residual enzyme activity was preserved, with BF11 maintaining 99% activity and VNH9 retaining 92%.

**Table 3:** Comparison of Modern and Traditional Purification Techniques: Efficiency, Cost, and Industrial Viability [50]

Aspect	Traditional Techniques	Modern Techniques
Efficiency	**Precipitation and Filtration**: Lower efficiency, longer processing times, lower yields and multiple steps required with **Purification Yield** 40-60%. Less effective in reaching high enzyme purity, may require multiple steps for purification. **Chromatography (Affinity & Gel Filtration)**: High purity but requires significant time and cost, with **Purification Yield** 60-90%.	**Reverse Micellar Systems (RMS)**: Higher recovery efficiency (15-fold improvement) with faster processing times.)80% recovery in 45 min; 52% recovery in 30–40 h (traditional method)(, with **Purification Yield** 80% (15-fold increase). **Immunopurification**: High specificity, near 99% residual activity, effective for precise enzyme isolation.High purity and quick isolation (efficient). with **Purification Yield** 92-99% residual activity
Cost	Lower initial costs, but longer processing times and lower yield increase operational costs	Higher initial cost due to specialized reagents (surfactants, antibodies), but cost-effective in the long run due to higher efficiency and lower processing time.
Industrial Application	Suitable for smaller-scale or less purity-critical industries, like certain food processing sectors.	Suitable for industries needing high-purity enzymes, such as biofuels, pharmaceuticals, and biotechnology.
Overall Viability	More suitable for smaller-scale production, but less viable for large-scale, high-purity enzyme production.	More cost-effective at industrial scale despite initial higher costs, with improved recovery and scalability.

## Lipase stability

### Challenges and opportunities

Lipase is an enzyme that plays a vital role in the hydrolysis of fats, essential in various industrial applications like food processing, pharmaceuticals, and biodiesel production. Lipase stability is a critical factor affecting its efficiency in these processes. Stability is influenced by several factors, including temperature, pH, solvents, and the presence of inhibitors or activators. Advances in techniques such as enzyme immobilization and protein engineering have significantly improved lipase stability, making it more suitable for industrial applications [51]. However, extreme conditions like sudden temperature fluctuations or the presence of non-polar compounds can significantly reduce enzyme stability, making it necessary to further enhance our understanding of enzyme-environment interactions [52].

### Factors affecting lipase stability

Studies have shown that lipase stability is highly dependent on its structural characteristics and the nature of the environment it operates in. For instance, some lipases exhibit high thermal stability, making them ideal for high-temperature applications. This includes industries such as food processing and pharmaceuticals, where enzymes are often exposed to extreme conditions. Understanding the structural compatibility of lipases with their environments is key to enhancing their stability [53]. Molecular mechanisms such as hydrogen bonds, ionic bonds, and hydrophobic interactions play crucial roles in maintaining the enzyme’s integrity. Changes in the ionization states or denaturation of these bonds can compromise the enzyme’s functionality [54].

### Immobilization techniques to enhance stability

Immobilization techniques are effective solutions to enhance lipase stability. Methods like enzyme attachment to solid supports or within gel matrices help improve enzyme resistance to harsh conditions such as high temperatures and organic solvents Table 4. Research has shown that immobilizing lipase using techniques like cross-linked enzyme aggregates (CLEAs) improves its operational stability and extends its shelf-life in industrial applications [55]. Furthermore, recent innovations in enzyme immobilization, such as using nanomaterials and microcapsules, have proven to increase enzyme stability even further in non-aqueous environments [56].

**Table 4:** Comparison of Free and Immobilized Lipases in Various Applications [57]

Aspect	Free Lipases	Immobilized Lipases
Activity	High initial activity, but prone to rapid loss under harsh conditions.	Slightly reduced initial activity but maintains stability over time.
Stability	Sensitive to temperature, pH, and organic solvents.	Enhanced stability under extreme conditions (e.g., high temperature, organic solvents).
Reusability	Single-use; not reusable, increasing production costs	Reusable in multiple cycles, reducing operational costs.
Supports Used for Immobilization	Not applicable	Natural supports (e.g., alginate, chitosan) and synthetic supports (e.g., silica, resins).
Applications	Suitable for one-time, small-scale reactions in laboratories	Ideal for large-scale industrial processes (e.g., biodiesel production, food industry).
Cost Efficiency	Lower initial cost but higher operational cost due to single-use nature.	Higher initial cost but more cost-efficient in long-term industrial applications.
Examples from Literature	Lipases from *Candida rugosa* used in free form for ester hydrolysis	Lipases immobilized on silica for biodiesel production, showing a 50% increase in productivity.

### Genetic engineering techniques for improving lipase stability and efficiency

1. Site-Directed Mutagenesis
  - **Mechanism**: This technique involves the targeted modification of specific amino acid residues in the enzyme’s active site or structural regions.
  - **Impact**: Enhances thermal stability, increases pH tolerance, and improves resistance to organic solvents by introducing stabilizing interactions such as hydrogen bonds, salt bridges, or disulfide bonds.
  - **Example**: A study on *C. antarctica* lipase B revealed that introducing disulfide bonds increased the enzyme’s thermal stability by 30% [58].

2. Directed Evolution
  - **Mechanism**: Mimics natural evolution by introducing random mutations and selecting variants with improved properties through high-throughput screening.
  - **Impact**: Produces lipases with enhanced stability and activity under extreme conditions without prior knowledge of the enzyme’s structure.
  - **Example**: Mutagenesis of *P. aeruginosa* lipase led to variants with a 5-fold increase in activity in organic solvents [59].

3. Fusion Protein Design
  - **Mechanism**: Fuses lipase with stabilizing domains or affinity tags to improve structural integrity and facilitate purification.
  - **Impact**: Results in increased stability and functionality in industrial processes.
  - **Example**: Fusion of *Thermomyces lanuginosus* lipase with a cellulose-binding domain enhanced enzyme stability and substrate binding in biocatalytic processes [60].

4. Computational Protein Design
  - **Mechanism**: Uses computational tools to predict mutations that enhance stability and catalytic efficiency by analyzing enzyme structure and dynamics.
  - **Impact**: Enables rational design of lipase variants tailored to specific industrial applications.
  - **Example**: Computational redesign of *Burkholderia cepacia* lipase improved its catalytic efficiency for esterification reactions by 40% [61].

5. Codon Optimization
  - **Mechanism**: Adapts the codon usage of the lipase gene to match the host organism’s preferred codon bias, enhancing protein expression.
  - **Impact**: Results in higher yields of stable and active lipase for industrial production.
  - **Example**: Codon optimization of *Geobacillus sp.* lipase in *Escherichia coli* increased expression levels by 2.5-fold [62].

6. Insertion of Glycosylation Sites
  - **Mechanism**: Adds glycosylation sites to the enzyme to improve solubility and structural stability under harsh conditions.
  - **Impact**: Protects the enzyme from degradation and increases thermal tolerance.
  - **Example**: Engineering glycosylation sites in *A. oryzae* lipase resulted in a 50% increase in thermal stability [63].

### Effect of organic solvents on lipase stability

The interaction between lipase and organic solvents is an important area of research for enhancing enzyme stability. Some lipases show high tolerance to organic solvents, making them suitable for non-aqueous systems. The appropriate choice of solvents can increase lipase stability and improve its catalytic efficiency in reactions like esterification and transesterification. Recent studies suggest that enzymes engineered to have a more rigid structure or altered surface hydrophobicity are less susceptible to denaturation in the presence of organic solvents [64].

### Future challenges and innovations

Despite significant progress in enhancing lipase stability, many challenges remain. A deeper understanding of the molecular mechanisms influencing lipase stability is crucial for designing enzymes that perform well in a wide range of industrial applications. Computational modeling and structural design are expected to further push the boundaries of lipase stability, offering solutions to the existing challenges. Furthermore, the integration of artificial intelligence and advanced predictive models could be key to designing more stable enzymes and optimizing their performance in industrial processes [65].

## Global impact of lipases on biotechnology

Lipases are widely used in biotechnology, especially in the production of biofuels, pharmaceuticals, and food processing. Their ability to catalyze reactions involving fats and oils has led to innovations in these fields, providing greener alternatives to traditional chemical processes. For instance, lipases are employed in the production of biodiesel, where they catalyze the transesterification of vegetable oils into biodiesel and glycerol. Additionally, lipases are involved in the synthesis of various pharmaceuticals and specialty chemicals, offering specific and efficient reactions that reduce byproducts and waste .

Moreover, lipases contribute to the advancement of industrial biotechnology by enabling the development of more efficient, environmentally friendly processes. For example, their use in the food industry enhances the production of high-value products, such as dairy and bakery goods, by improving flavors and textures, reducing processing times, and increasing the shelf life of products [66].

## Global market and future trends in lipase biotechnology

1. **Market Data**: Include global market size estimates, such as current annual revenue figures for the lipase market and key regions showing the highest demand (e.g., North America, Europe, Asia).
2. **Future Projections**: Add information on the expected market growth, such as “the lipase market is expected to grow at a rate of X% annually from 2025 to 2030.”
3. **Future Challenges**: Mention potential future challenges for the market, like production costs, the need for enzyme effectiveness improvements, or environmental limitations in large-scale production .
4. **Emerging Trends**: Expand on promising applications such as **genetic engineering** to improve lipase production, and the use of **modern technologies** like **bioreactors** [67].

## Preclinical and clinical trials on lipases

1. **Preclinical Studies**: Animal models have shown lipases’ potential in cancer therapy and targeted delivery, with research focusing on improving stability and reducing degradation in the body [68].
2. **Clinical Trials**:
  **Phase I**: Early trials showed lipases are safe for humans, with minor side effects [69].
  **Phase II/III**: Lipase therapies have shown promise in treating cancer and metabolic diseases, but challenges like higher doses and stability remain [69].

3. **Future Perspectives**:
  **Gene Therapy**: Exploring lipase overexpression in cancer cells for targeted therapy.
  **Combination Therapies**: Lipases may enhance traditional therapies like chemotherapy.
  **Optimized Delivery**: Focus on improving stability, targeting, and bioavailability [70].

## Clinical challenges in lipase therapy

1. **Regulatory Barriers**: Lipase-based therapies face stringent approval processes, requiring extensive data to ensure safety and efficacy. Standardization of production is also a key challenge.
2. **Immunogenicity**: Recombinant lipases may trigger immune responses, leading to allergic reactions. Humanizing lipases can help reduce immune recognition.
3. **In vivo Stability**: Lipases may be degraded by proteases in the body, reducing their effectiveness. Targeted delivery methods are being explored to enhance stability [71].

**Using lipases instead of chemical alternatives involves several economic considerations**:

**Costs**:

- **Production**: Lipase production can be expensive due to fermentation, purification, and enzyme stabilization.
- **Scale-Up**: Transitioning to large-scale production can be costly and complex.

**Benefits**:

1. **Lower Environmental Impact**: Lipases work under mild conditions, reducing energy use and harmful by-products.
2. **Cost Savings**: Less need for harsh chemicals, which lowers chemical costs.
3. **Higher Efficiency**: Lipases offer more selective and efficient reactions, reducing waste and improving yields.
4. **Reusability**: Immobilized lipases can be reused, lowering costs over time.
5. **Sustainability**: Their use aligns with the demand for greener, eco-friendly processes, improving market appeal [72].

## Lipase as antibiofim

**Fig. 4:**
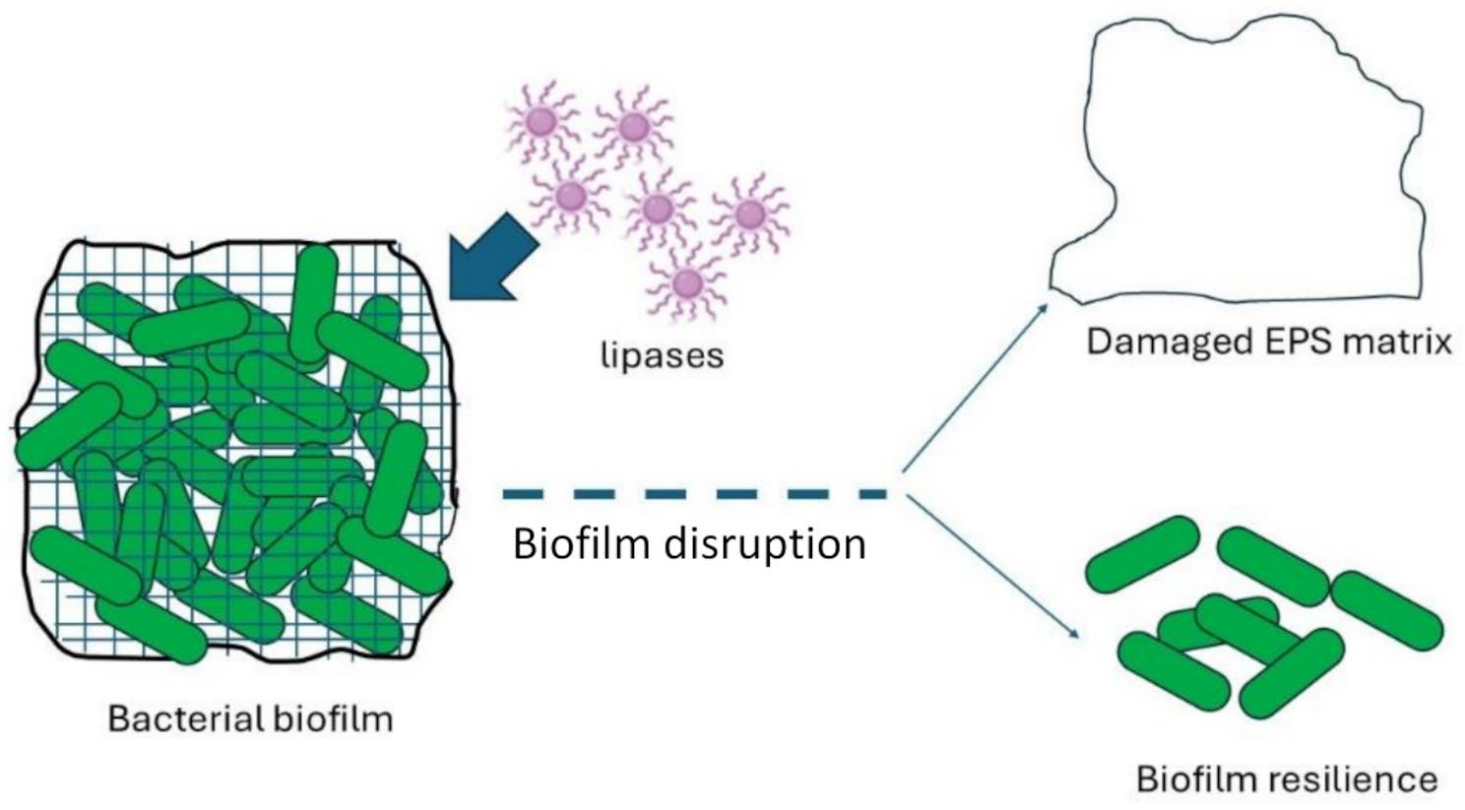
A simple schematic diagram clarifies the effect of lipase enzyme on biofilm

Because the pores are connected, porous biomaterial is the chosen implant material. The pore structure of these biomaterials increases the risk of biofilm-induced infection. Despite the fact that implant biofilm is responsible for 80% of human infections [73], no naturally occurring medication is marketed to treat it. In the current work, the Langmuir-Blodgett deposition technique was used to transfer glutaraldehyde cross-linked lipase onto an activated porous polycaprolactam surface. The surface properties, slimicidal, antibacterial, thermostable, and biocompatible characteristics of the lipase were examined. There was a 20% increase in the activity of the covalently crosslinked lipase when compared to its free form. Comparing the covalently crosslinked lipase to its free form, there was a 20% increase in its activity. At temperatures as high as 100 °C, the immobilized surface remained stable and active due to its thermostability. Compared with uncoated polycaprolactam (UP), lipase immobilized polycaprolactam (LIP) resulted in a 2 and 7 times reduction in carbohydrates and a 9 and 5 times reduction in biofilm protein of *Staphylococcus aureus* and *Escherichia coli*, respectively. Comparing LIP to UP, there were four times less live bacterial colonies observed. As demonstrated by AFM, fluorescence microscopy pictures, and the quantity of lactate dehydrogenase generated, lipase attacked the bacterial cell wall, causing it to die. Indicating that LIP was biocompatible, it permitted the proliferation of almost 90% of 3T3 cells. The fact that LIP exhibits antimicrobial property at the air-water interface to hydrophobic as well as hydrophilic bacteria along with lack of cytotoxicity makes it an ideal biomaterial for biofilm prevention Fig. (4) in implants [74].

## Lipase in cancer treatment

**Fig. 5:**
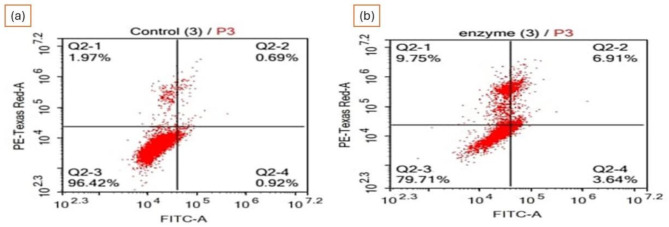
(**A**) flow cytometry data of (**a**) control Hep-G2 cells and (**b**) microbial lipase-treated cells showing move of the affected cells towards late apoptosis. (Q1) denotes necrosis phase, (Q2) refers to late apoptosis stage, (Q3) means normal cells and (Q4) shows early apoptosis stage

The risk of liver, colon, breast, pancreatic, and prostate cancers may be attributed to a reduction in physical activity and a high calorie diet [75]. As a result, the serum’s triglyceride (TG) concentrations showed signs of colorectal and pancreatic malignancies, as well as precancerous lesions. It is also known that the lipoprotein lipases (LPL) hydrolyze plasma TG [76]. It is anticipated that the deletion of a putative tumor suppressor gene on the short arm of chromosome 8 in humans will either cause or facilitate the development of hepatocellular carcinoma. The FISH research provides proof that an LPL deficit contributes to the development of prostate cancer [77]. Additionally, our work on the effect of microbial lipase from *P. aeruginosa* showed promotion of apoptosis in HEpG-2 cancer where the percentage of early apoptotic cells augmented from 2.27 to 30.37%, recording more than 13-folds increase, whereas the late apoptotic cells improved from 0.69 to 7.24%, indicating more than 10-fold increase (*in press*). Moreover, necrotic cells increased from 0.71 to 3.68% recording more than 5-fold increase, after handling the cells with microbial lipase suggesting anticancer action of microbial lipase on liver cancer cells via stimulation of both apoptosis as well as necrosis Fig. (5).

Other cancer-prone genes were also removed from the human chromosomal short arm; these genes are in charge of mitochondrial tumor suppressor 1 (MTUS1), liver cancer 1 (DLC1), and breast cancer 2 (DBC2) [85]. Accordingly, the LPL gene loss on this chromosome shifts the closest genes linked to cancer into question, and their combined impact in promoting carcinogenesis is documented. Individuals with many cancer types have cachexia, which is characterized by a loss of skeletal muscle and adipose tissues, lipid metabolism, and triglyceride hydrolysis. Cachexia is defined as weakening and wasting of the body as a result of profound chronic sickness. In the metabolism of lipids and lipoproteins, LPL has an effect on triglycerides (TG) and monoglycerides (MG).

LPL modulators, including tumour necrosis factor (TNF)-α and interleukins (IL-1, IL-6), are activated by cachexia, which prevents LPL from doing its job and leads to a sharp reduction in the build-up of fatty tissues [77].

- **Lipases induce apoptosis in cancer cells through several mechanisms**:
  1. **Lipid Metabolism**: Lipases hydrolyze lipids, producing bioactive molecules like ceramides, which activate apoptosis pathways by affecting mitochondrial function and caspases.
  2. **Mitochondrial Pathway**: Lipases disrupt mitochondrial membranes, releasing cytochrome c and triggering the caspase cascade, promoting cell death.
  3. **Caspase Cascade**: Lipases can activate both intrinsic (mitochondrial) and extrinsic (death receptor) pathways, initiating apoptosis through caspase activation.
  4. **Endoplasmic Reticulum (ER) Stress**: Altering lipid metabolism causes ER stress, triggering the unfolded protein response (UPR) that leads to apoptosis [78].

- **Integration with Therapies**:
  1. **Chemotherapy**: Lipase-induced apoptosis can sensitize cancer cells to chemotherapy by increasing ROS and improving drug uptake.
  2. **Targeted Therapies**: Lipases can enhance drug delivery by modifying cell membranes and help overcome drug resistance.
  3. **Immunotherapy**: Lipases may modulate the tumor microenvironment, improving the effectiveness of immunotherapies like PD-1 inhibitors.
  4. **Gene Therapy**: Overexpressing lipases in tumor cells could selectively induce apoptosis, sparing normal cells [78].

## Role of lipases in biosensors

A rising field of interest is the employment of lipases in biological tests and biosensors. Lipases have a broad substrate specificity and are more widely available commercially, which makes them widely used. There are two applications for lipases in biosensors Sandoval et al., [79] state that they can be used as enzymatic substrates or inhibitors.

**Fig. 6:**
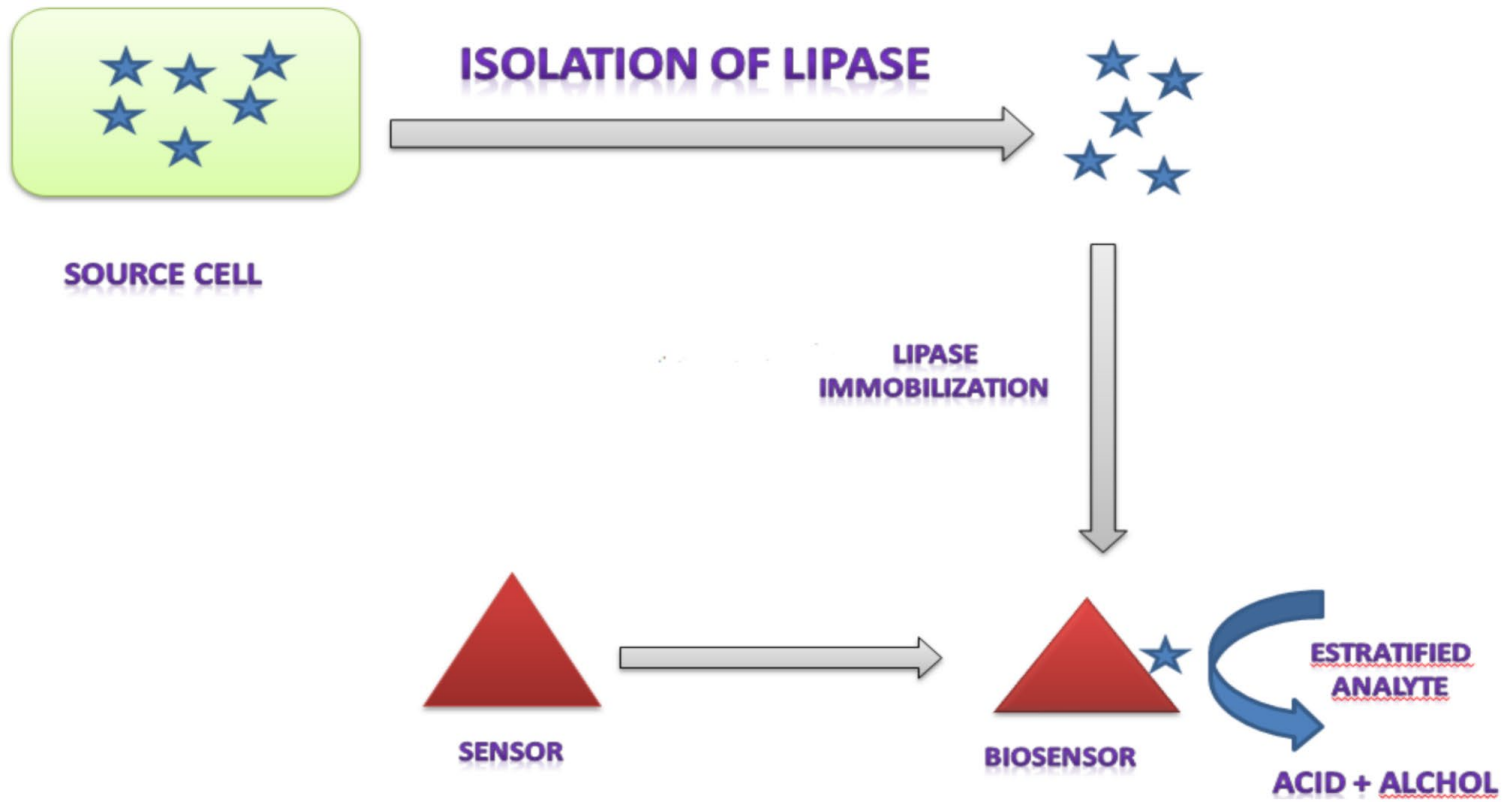
Lipase isolation and acting as biosensor [81]

In analytical techniques used in various fields like environmental science, the food industry, biodegradable polymers, oleochemicals, and as diagnostic instruments as in Fig. (6) to measure triglyceride and cholesterol levels in blood samples, the enzymatic biosensors of lipases play a critical role [80]. Various inhibitors can impede the activity of lipases. Several lipase inhibitors from diverse sources have been reported in several recent research. As per [3] lipase from *B. cereus* gets inhibited by cetyltrimethyl ammonium bromide. Lipase from *Yarrowia lipolytica* is inhibited by cadmium-II, but lipase from *Mycobacterium tuberculosis* is inhibited by cobalt-II.

- **Lipase Applications as biosensor.**
  1. **Commercialized Devices**:
    **Food Quality**: Lipase-based biosensors monitor freshness by detecting rancidity in oils and foods.
    **Environmental Monitoring**: Used to track oil degradation in environmental pollutants like oil spills.
  2. **Accuracy and Validation**:
    **Accuracy**: Lipase sensors are precise, offering real-time measurement of fat content and pollutants.
    **Validation**: Sensors are validated using standard methods to ensure reliable, reproducible results [81].

## Lipase in cosmetics industry

The Cosmetics Industry Cosmetics are the general term for commercial personal care products. Apart from natural soaps, solvents, and fragrance ingredients, the cosmetic chemicals market in the US, Europe, and Japan was valued at roughly USD 6.8 billion in 2008. According to their uses, cosmetics have been divided into a number of categories recently. These categories include skin care, hair care, toiletries, ornamental cosmetics, mouthwash, and toothpaste [82].

Due to their significance as active components in cosmetic formulations and as biocatalysts in the manufacture of particular chemicals used in cosmetics, lipases are regarded as the most important enzymes in the manufacturing of various types of cosmetics. Cosmetics that target cellulite, remove debris and small flakes of dead corneous skin, and clean the skin superficially are the principal uses of active lipases [82]. Lipases are frequently used as active components in cosmetics by encasing them in nanoparticles [83].

Lipases are used to encapsulate active ingredients like vitamins and peptides, improving their stability and controlled release in products like anti-aging serums. They also help produce emulsifiers and surfactants for creams, lotions, and shampoos, enhancing texture and stability [84].

Lipase encapsulation particles consist of an oily dissipating media mixed with a core substance containing the lipases. Aluminum tristearate or di-is used to stabilize this. Agar serves as a supplementary ingredient [82].

Hollow spheres made of inorganic silica have been suggested by Fujiwara and Nakahara [85] as potential materials for lipase encapsulation. According to a study by Miguez et al. [86]. talked about the best possible enzymatic production of a cosmetic ester (decyloleate), which was aided by a handmade biocatalyst created by physically attaching lipase (from *Thermomyces lanuginosus*) to silica from rice husk amino functionalizations. Their findings demonstrated 87% ester conversion in a solvent-free system, and the biocatalyst continued to function after 8 batches. Lipases are applied in cosmetic formulations using a variety of approaches, depending on the application method that is recognized for a specific interest in industrial processes.

## Lipase in food industry

Food industry lipases are now a crucial component of the contemporary food chain. The demand for microbial lipases has increased globally due to the diverse uses of lipases in the food industry. Microbial lipases have a critical role in the processing of meals containing fat, the creation of other dairy products’ flavors, the production of linear meat, the customization of vegetable oils, and cheese flavor enhancement [87]. Because lipases exhibit some enzymatic processes at mild circumstances, they are expected to play a significant role in the edible oil sector. Likewise, lipases alter tastes and scents by creating alcohol and short-chain fatty acid esters [87].

The dairy products sector uses lipases from *Pseudomonas* species, *Bacillus* species, *Penicillium* species, *Rhizopus* species, and Mucor species in various operations. Microbial lipases are frequently utilized for the hydrolysis of lipids in milk, which affects the length of the fatty acid chain, increases the flavor of cheese, and, in particular, aids in the development of soft cheese [88, 89]. Good grade cheese has recently been prepared using single microbial lipases or mixes of microbial lipases [90].

Additionally, microbial lipases have been widely employed to improve the flavor of rice, add fragrance to apple wine, and alter soybean milk [91, 92]. Lipases are also utilized in biolipolysis, a process that reduces meat’s fat content by adding lipases [91–93].

## Lipase in detergent industry

Detergents contain lipases to improve their cleaning power. Because they are environmentally benign, effective at low temperatures, and do not lose their activity after stain removal, lipase-based detergents outperform synthetic detergents in terms of cleaning capabilities [94]. Lipase-based detergents are designed to remove fat soil from fabrics by combining lipases with an ionic or nonionic surfactant. Liquid laundry detergents that employ encapsulated enzymes are becoming more and more popular. For cold washing, cold-active lipase detergents are used to lessen fabric deterioration and energy consumption [95, 96].

In the detergent business, microbial lipases are commonly utilized; alkaline yeast lipases are favored over fungal and bacterial lipases [94]. In their study, Maharana and Singh isolated *Psychrotolerant Rhodotorula* sp. Y-23 and used it to manufacture lipase by a plate assay and submerged fermentation. They also outlined an ideal procedure for producing the most lipase possible at 15 °C using palmolein oil (5% v/v). They also reported lipase production inducers (galactose, potassium nitrate [KNO3], and manganese chloride [MnCl_2_]), assessed the enzyme’s tolerance, and came to the conclusion that lipase was compatible with detergents that were sold in stores. Lipid breakdown is increased when lipases from the *Psychrotolerant* yeast *Rhodotorula* sp. Y-23 are added to various detergents. The addition of lipases from psychrotolerant yeast Rhodotorula sp. Y-23 to different detergents augments lipid degradation, making it a potential candidate for use in the detergent [97].

Due to differences in the ideal pH and temperature within these families, lipases derived from microbial sources were divided into eight groups according to their unique characteristics [98]. A novel strain of *Pseudomonas helmanticensis* HS6 was identified by Phukon et al. [99] for the generation and characterisation of lipase intended for use in the detergent sector. According to [100–102], they reported an 18.78-fold increase in the synthesis of pure lipase, which exhibited optimal activity at 50 ◦C and a pH of 7. It was found that an enzyme could be active throughout a broad range of temperatures (5–80 ◦C) and pH. Additionally, after being incubated with commercial detergents for 3 h, the lipase from *P. helmanticensis* HS6 maintained residual activity of 40–80%. which suggests its suitability for applications in the detergent industry.

## Lipase in medical applications

When *Penicillium* extendus was used to separate the intermediate, Dai and Xia [103] discovered that lipase’s enantioselectivity was greater than 100–fold. One of this technology’s benefits is the ability to separate ibuprofen isomers. The lipase of *Rhodothermus marinus* DSM 4252 broke down the immobilized ibuprofen ester to produce (S)- enantiopure ibuprofen [104].

The levels of phospholipid, cholesterol, and triglycerides in patient blood samples are measured using lipase-based sensors as diagnostic instruments [105]. Likewise, lipase derived from staphylococcal strains has been employed in the production of antioxidants such eugenyl benzoate and ethanol-acetate [106].

## Role of lipases in biodiesel production

Lipases’ function in the production of biodiesel to create biodiesel (mono-fatty acid alkyl esters, or FAAE) from fats and oils, lipases can be utilized in the esterification and transesterification processes [107, 108]. While lipase’s use in the previously listed industries (food, leather, textile, etc.) is well known, its application in the biodiesel synthesis process is still in its infancy. The serine (Ser) nucleophile in the lipase’s active center engages in a charge relay system with histidine (His), glutamic acid (Glu), or aspartate (Asp) residues for the nucleophilic attack with alcohol on the ester bonds in the transesterification reaction when triglycerides are substrates in a nonaqueous medium.Two products come from this reaction: an intermediate product and an acetylated enzyme. Diarylglycerols are a result of triacylglycerol’s conversion to an alkyl ester with a single carboxyl group. As a result, three mono fatty acid alkyl esters (biodiesel) are produced, and monoacylglycerol is created, which releases the glycerol backbone in triacylglycerol and all phases of this reaction. After then, more alcohol is added for stabilization [109].

Lipases have been utilized to prepare biodiesel from oils and fats for the past thirty years. However, large-scale biodiesel manufacturing is catalyzed by various chemicals, such as acids and bases. Lipases must be widely used in the manufacturing of biodiesel if it is to remain a fuel that emits no carbon dioxide in the future. At the moment, several researchers are producing biodiesel using free lipase [110, 111]. Nonetheless, the majority of research advises using lipase’s immobilized form for this purpose [112].

A number of processes are optimized with immobilized lipase in mind for the manufacture of biodiesel. The selection of microbial lipases for biodiesel production is contingent upon their origin and formulation [111, 112].

- **Biodiesel Production: Lipases vs. Chemical Catalysts**.
  1. **Tolerance to High Free Fatty Acid Content**:
    **Lipase**: Effective with oils high in free fatty acids, using *T. lanuginosus* lipase in a two-phase system.
    **Chemical Catalysts**: Require pre-treatment to remove free fatty acids.
  2) **Efficiency and Productivity**:
    **Lipase**: Fast conversion, achieving nearly complete biodiesel production in 6 h.
    **Chemical Catalysts**: Faster initial reactions but lower efficiency due to by-products.
  3) **Technical Challenges**:
    **Lipase**: Bottleneck in converting DAG and MAG; overcome by silica particles improving emulsion structure.
    **Chemical Catalysts**: Challenges in heat and mass transfer, leading to by-products.
  4) **Environmental Impact**:
    **Lipase**: Eco-friendly, reducing emissions and using waste oils.
    **Chemical Catalysts**: Produce harmful by-products and are energy-intensive [113].

## Role of lipases in bioremediation

Petroleum, pesticides, fertilizers, plastics, and other hydrocarbon-containing materials are examples of xenobiotic molecules that have a continuous negative impact on our natural environment. To remove these pollutants, a number of strategies have been developed. These methods, however, are not efficient or eco-friendly. Enzyme-based bioremediation is an easily adjustable technique that removes these dangerous elements from our natural ecosystem in a more gentle manner than chemical or physical treatments. However, these enzymes’ limited use in bioremediation is due to production challenges and exorbitant costs. As a result, using microbial enzymes for bioremediation is becoming more and more significant on a global scale. Furthermore, microbial enzymes are better able to convert pollutants into benign compounds and reduce pollution in the environment. Recently, Jacob et al. [114].

Similar to this, it is imperative to address the growing amounts of micro-nanoplastics (MNPs) in natural ecosystems since MNPs have a detrimental effect on the wellbeing of all living things. Numerous studies have documented the application of fungal strains like Aspergillus clavatus and bacterial strains resistant to plastic, such as *B. amyloliquefaciens* 1 and 2, in disposal locations for trash. These bacteria break down plastic in several ways, either directly via the employment of microbial enzymes like lipases or indirectly through the use of plastic as a carbon source. Lipases are produced by bacterial and fungal species such as *P. fluorescens*,* P. aeruginosa*,* Penicillium simplicissimum*,* C. cylindracea*,* and Rhizopus delemar*. These species have been shown to be capable of breaking down MNPs [115–117].

**Bioremediation: Field Use and Degradation Rates**.

**Fig. 7:**
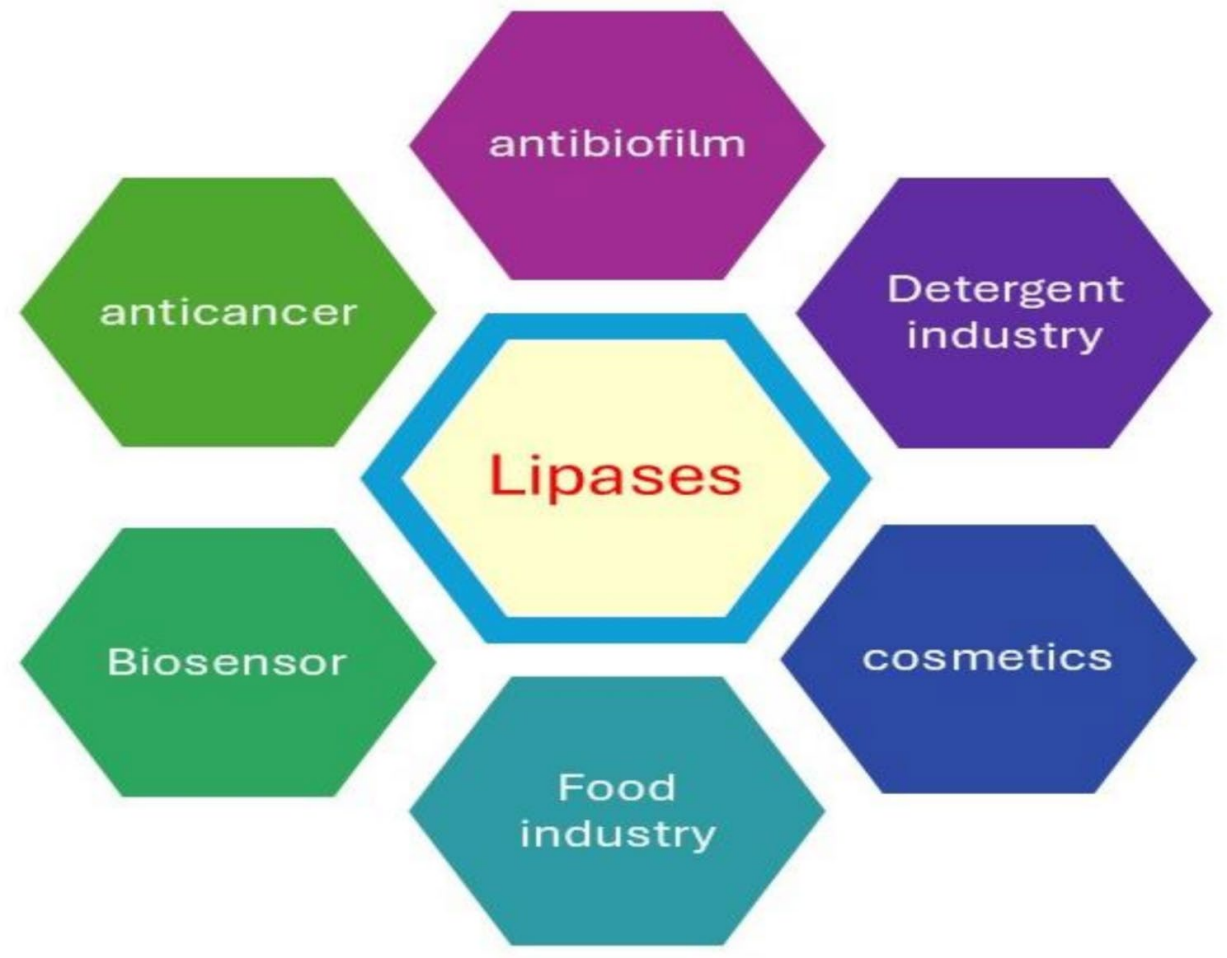
Applications of lipase in health and industry [25]

1. **Plastic Degradation**:
  **Microplastics**: Microorganisms like *Ideonella sakaiensis* have been used to degrade microplastics in marine environments. Degradation rates range from 30 to 60% over weeks.
  **Nanoplastics**: Bacteria such as *Pseudomonas* can degrade nanoplastics in river environments at rates of 30–60% over 2–3 weeks.

2. **Other Pollutants**:
  **Oil Spills**: Microorganisms like *Alcanivorax borkumensis* degrade oil at 1–3% per day.
  **Heavy Metals**: *Bacillus* species can remove up to 80% of lead and cadmium in contaminated water.

3. **Degradation Rates**:
  **Microplastics**: 10–70% degradation in weeks to months.
  **Nanoplastics**: 30–60% degradation in weeks.
  **Oil/Heavy Metals**: Hydrocarbons degrade at 2–5% per day [117–119]. Different applications of lipases in health as well as industry are summarized in Fig. 7.

## Integration of lipases into biorefinery and circular bioeconomy models

1. **Biorefinery Applications**: Lipases convert waste oils and fats into biodiesel, fatty acids, and other valuable chemicals, supporting waste-to-value conversion and the production of bio-based alternatives to petroleum products.
2. **Circular Bioeconomy**: Lipases enable recycling and upcycling of oils and plastics into reusable monomers or biofuels, contributing to a closed-loop system that minimizes waste and conserves resources.
3. **Biodegradable Products**: Lipase-based processes support the production of biodegradable plastics and surfactants, reducing reliance on non-degradable plastics [109, 112, 120].

## Challenges and strategies in scaling lipase production to meet diverse industrial demands

1. **Optimizing Fermentation**: Improving conditions for microbial growth and productivity, and using alternative substrates like agro-industrial waste.
2. **Improving Enzyme Stability**: Using techniques like enzyme immobilization and protein engineering to enhance stability under industrial conditions.
3. **Efficient Downstream Processing**: Streamlining purification and recovery methods to reduce costs and increase yield.
4. **Cost Reduction**: Utilizing low-cost substrates and improving reactor efficiency to lower production costs.
5. **Automation**: Integrating real-time monitoring and automated systems to ensure consistent quality and reduce human error [121–123].

## Conclusion and future prospects

Microbes have been proven to be an efficient and versatile microbial cell factory for the production of lipases, offering high enzyme yields and robust catalytic properties. Advances in fermentation strategies, purification techniques, and enzyme engineering have further enhanced the utility of microbial lipases in various industrial sectors, including food, pharmaceuticals, cosmetics, and biofuels. Additionally, its potential applications in bioremediation, drug delivery, and medical diagnostics demonstrate the enzyme’s growing relevance beyond traditional uses.

Looking ahead, future research should focus on optimizing production using sustainable substrates, such as agro-industrial by-products, to reduce costs and promote environmentally friendly processes. Further exploration of enzyme immobilization techniques and protein engineering could enhance the stability and specificity of microbial lipase, broadening its range of applications. The integration of lipase production with biorefineries and circular bioeconomy models also holds promise for improving process efficiency and sustainability.

Moreover, advances in synthetic biology and systems biology approaches could enable the engineering of microbial consortia or synthetic pathways for more efficient lipase production. Addressing biosafety challenges through genetic modifications and regulatory frameworks will be crucial to facilitating the wider acceptance of microbial lipases in both industrial and biomedical settings. With continued interdisciplinary efforts, microbes can play a pivotal role in shaping the future of enzyme technology and sustainable development.

## Data Availability

No datasets were generated or analysed during the current study.
